# Myocardial involvement is not rare in anti-melanoma differentiation-associated gene 5 antibody-positive dermatomyositis/clinically amyopathic dermatomyositis: a retrospective study

**DOI:** 10.3389/fimmu.2022.928861

**Published:** 2022-08-02

**Authors:** Shuang Zhou, Jinzhi Lai, Chanyuan Wu, Yongtai Liu, Yingxian Liu, Jiuliang Zhao, Dong Xu, Xinping Tian, Mengtao Li, Yan Zhao, Yining Wang, Qian Wang, Xiaofeng Zeng

**Affiliations:** ^1^ Department of Rheumatology and Clinical Immunology, Chinese Academy of Medical Sciences & Peking Union Medical College, National Clinical Research Center for Dermatologic and Immunologic Diseases (NCRC-DID), Ministry of Science & Technology, State Key Laboratory of Complex Severe and Rare Diseases, Peking Union Medical College Hospital (PUMCH), Key Laboratory of Rheumatology and Clinical Immunology, Ministry of Education, Beijing, China; ^2^ Department of Cardiology, Peking Union Medical College Hospital, Chinese Academy of Medical Sciences & Peking Union Medical College, Beijing, China; ^3^ Department of Radiology, State Key Laboratory of Complex Severe and Rare Diseases, Peking Union Medical College Hospital, Chinese Academy of Medical Sciences & Peking Union Medical College, Beijing, China

**Keywords:** anti-melanoma differentiation-associated gene 5 antibody, dermatomyositis, clinically amyopathic dermatomyositis, myocardial involvement, interstitial lung disease

## Abstract

**Objectives:**

Studies concerning myocardial involvement (MI) in patients with anti-melanoma differentiation-associated gene 5 antibody-positive dermatomyositis/clinically amyopathic dermatomyositis (anti-MDA5 Ab+ DM/CADM) are scarce. We aimed to characterize MI in our anti-MDA5 Ab+ DM/CADM cohort and to investigate its association with prognosis.

**Methods:**

In this single-center retrospective study, anti-MDA5 Ab+ hospitalized DM/CADM patients who underwent transthoracic echocardiography (TTE) were enrolled. Myocardial involvement was diagnosed according to abnormal cardiac structure and function detected by TEE. Clinical features and cardiac examination findings of patients with MI were analyzed. Clinical features, laboratory findings, complications, and treatments were compared between MI and non-MI, deceased, and survival patients. Logistic regression analysis was used to explore the independent risk factors for the occurrence of MI and prognostic factors for these patients.

**Results:**

Seventy-six hospitalized patients with anti-MDA5 Ab+ DM/CADM were enrolled. Twelve (15.8%) patients were diagnosed with MI. Of the 12 patients, three underwent cardiac magnetic resonance imaging (CMR) and late gadolinium enhancement (LGE) were noted for them. TEE revealed that eight (66.7%) patients had left atrial and/or ventricular enlargement, three (25.0%) had cardiac hypertrophy, six (50.0%) had diffuse ventricular wall dyskinesia, and seven (58.3%) had diastolic dysfunction. Six (50.0%) patients with MI developed heart failure (HF) during treatment. Of the 12 patients, one patient died of HF caused by myocarditis, three died of infection, and four died of exacerbation of rapidly progressive interstitial lung disease (RP-ILD). Logistic regression analysis revealed that dysphagia (OR 3.923, 95% CI 1.085, 14.181), NT-proBNP >600 pg/ml (OR 18.333, 95% CI 1.508, 222.875), and increased peripheral white blood cells (OR 1.201, 95% CI 1.003, 1.438) were risk factors for the occurrence of MI, but plasma albumin (OR 0.892, 95% CI 0.796, 0.999) was a protective factor. Both MI (OR 5.984, 95% CI 1.174, 30.496) and RP-ILD (OR 11.875, 95% CI 2.796, 50.411) were independent risk factors for the mortality of these anti-MDA5 Ab+ DM/CADM patients.

**Conclusion:**

Myocardial involvement is not rare and is an independent poor prognostic factor of anti-MDA5 Ab+ DM/CADM patients. Cardiac abnormality screening is necessary for them.

## Introduction

Idiopathic inflammatory myopathy (IIM) is a heterogeneous group of autoimmune diseases, characterized by muscle weakness and extramuscular involvements, including specific skin lesions, interstitial lung disease (ILD), cardiac involvement, arthritis, and sometimes an association with malignancy. At present, myositis-specific autoantibodies (MSAs) and myositis-associated autoantibodies (MAAs) have been widely used to help in disease diagnosis, characterize and subcategorize patients, and predict prognosis. The anti-melanoma differentiation-associated gene 5 (anti-MDA5) antibody was firstly reported in 2005 as an anti-clinically amyopathic dermatomyositis-140 (anti-CADM-140) antibody (1). After that, RNA helicase encoded by MDA5 was identified as the major autoantigen of anti-CADM-140 in 2009 (2). Patients with a positive anti-MDA5 antibody (anti-MDA5 Ab+) are typically characterized by the presence of skin ulcer and rapidly progressive interstitial lung disease (RP-ILD), whereas the manifestations of myositis are frequently slight or absent (3, 4).

Cardiac involvement is as crucial a complication of IIM as ILD and is a prognostic factor for unfavorable outcome. The frequency of cardiac involvement in patients with IIM varies between 9% and 72% (5). Documented cardiac manifestations in IIM patients are diverse including myocardial ischemia, arrhythmias, conduction defects, cardiomyopathies, pericardium diseases, and pulmonary hypertension (5), whereas reports of cardiac involvement in anti-MDA5 Ab+ dermatomyositis/clinically amyopathic dermatomyositis (DM/CADM) patients are scarce. In the past decades, only three cases of anti-MDA5 Ab+ DM patients with severe myocardial defects have been reported (6–8). A recent study revealing distinctive electrocardiography (ECG) changes in anti-MDA5 Ab+ DM/CADM patients (9) further indicates cardiac involvement of anti-MDA5 Ab+ DM/CADM patients. However, the prevalence of myocardial involvement (MI) in patients with anti-MDA5 Ab+ DM/CADM and its impact on prognosis remain unclear. A former Japanese study observed that in their anti-MDA5 Ab+ DM/CADM cohort, all of the deaths occurred within the first 6 months of DM/CADM diagnosis and none of the survivors suffered relapsing of RP-ILD (3). Thus, it is worth exploring whether MI is another critical factor affecting the prognosis of anti-MDA5 Ab+ DM/CADM patients. Precise clinical characteristics of anti-MDA5 Ab+ DM/CADM patients remain to be elucidated, which are necessary to improve the management of this life-threatening severe disease.

Therefore, in this study, we retrospectively analyzed MI in a large group of hospitalized anti-MDA5 Ab+ DM/CADM patients to identify the risk factors for occurrence of MI and to explore whether MI is a prognostic factor in these patients.

## Materials and methods

### Patients

This study was approved by the medical ethics committee of the Peking Union Medical College Hospital (approval number: S-K1997). Due to the retrospective nature of this study, it did not influence doctors’ treatment decisions or required additional examinations. Therefore, patient informed consent was waived. We retrieved medical records of adult patients (≥18 years old) who were hospitalized in Peking Union Medical College Hospital from January 2015 to September 2021. Patients included in this study were diagnosed with DM or CADM with anti-MDA5 Ab+. The diagnosis of DM fulfilled the criteria of Bohan and Peter (10). The diagnosis of CADM was based on the criteria suggested by Sontheimer (11). Patients would be excluded for the following reasons: 1) DM/CADM overlapped with other connective tissue diseases; 2) hospitalization for reasons unrelated to DM/CADM or its complications; 3) loss of follow-up within 1 month after discharge.

### Methods

Clinical data were collected by reviewing the electronic medical record system. We extracted detailed information on patient demographics, clinical and laboratory findings, imaging reports, treatment, and outcomes. Myocardial involvement would be diagnosed when myocardial defects were confirmed by transthoracic echocardiography (TTE) or cardiac magnetic resonance imaging (CMR) directly, or when TTE suggested abnormalities in cardiac structure or function that the cardiologists attributed to DM rather than secondary to other factors such as age, arrhythmias, hypertension, coronary heart disease (CHD), and other myocardiopathies. Isolated situations including arrhythmia, conduction abnormality, pericardium diseases, pulmonary hypertension, and CHD considered to be secondary to DM but without supportive evidence of myocardial defects by TTE or CMR would be excluded either. ILD was diagnosed by chest computed tomography (CT). The criteria for diagnosis of RP-ILD in this study were revised according to the diagnostic criteria for acute exacerbation of idiopathic pulmonary fibrosis (AE-IPF) (12, 13), which were as follows: 1) previous or concurrent diagnosis of ILD; 2) progressive dyspnea within 1 month; 3) chest CT findings with newly developed bilateral ground-glass opacification and/or consolidation not fully explained by left heart failure or fluid overload, regardless of the presence of infection. The diagnosis of bacterial, viral, and fungal infection was made according to the comprehensive decision by clinicians based on symptoms, imaging changes, laboratory abnormalities, and microbiological findings of sputum, bronchoalveolar lavage fluid (BALF), and blood. The hospital mortality in this study referred to the death occurred in the period of hospitalization or within 2 weeks after discharge if patients left the hospital against medical advice.

### Statistical analysis

Continuous variables were described as mean ± SD if normal distribution and median (interquartile range) if skewed distribution. Comparison of normally distributed continuous variables was performed by independent sample *t* test. Comparison of skewed continuous variables was performed by Mann–Whitney U test. Categorical variables were presented as frequencies and percentages. Comparison of categorical variables was performed by chi-square test. Logistic regression analysis was performed to identify association factors with the occurrence of MI and to identify independent associated factors for mortality of anti-MDA5 Ab+ DM/CADM patients. Results of univariate and multivariate logistic regression analyses were presented as an OR with 95% CI. *p*-values < 0.05 were considered statistically significant. All statistical analyses were performed using SPSS version 23.0 (Chicago, IL, USA).

## Results

### Clinical characteristics of patients with myocardial involvement

A total of 100 hospitalized patients were diagnosed as anti-MDA5 Ab+ DM/CADM. Among them, 90 (90.0%) patients underwent myocardial enzyme detection and 97 (97.0%) patients underwent ECG on admission. Seventy-six (76.0%) patients also underwent TTE during hospitalization. Three of these 76 patients performed CMR as well during the period of treatment. The overview of cardiac examinations taken by these 100 patients is noted in **Supplementary Table S1**.

**Figure 1:**
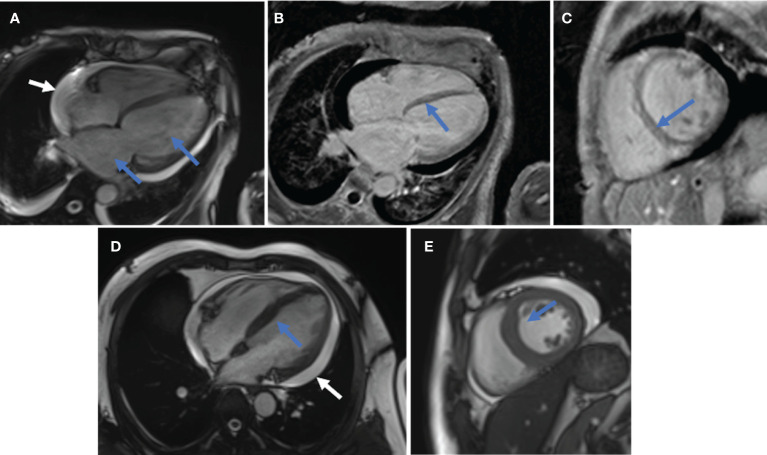
Representative CMR images. **(A–C)**, patient 2. **(A)**, Enlarged left atrium and left ventricle (blue arrow); pericardial effusion (white arrow). **(B, C)**, Linear LGE in the basal-mid segment of ventricular septum (**B** in long-axis view, **C** in short-axis view). **(D, E)**, patient 4. **(D)**, Thickened ventricular septum (blue arrow) and pericardial effusion (white arrow) in long-axis view. **(E)**, Thickened ventricular septum (blue arrow) in short-axis view.

Twelve (15.8%) of these 76 patients were finally identified with MI. Five of these 12 patients (patients 2, 3, 4, 5, and 7) were initially diagnosed with TTE or CMR directly. However, a detailed review of the medical records of 76 patients revealed that another seven patients with abnormalities in TTE findings could be attributed to MI of DM/CADM. Myocardial enzymes, ECG, and TTE were available for all 12 patients. These findings together with demographic and baseline clinical features are summarized and shown in **Table 1**. The median age of these 12 patients was 55.5 years, with a 1:1 male to female ratio. The median disease course was 3 months. None of the 12 patients had hyperlipidemia or CHD, but two (16.7%) patients had hypertension, two (16.7%) had diabetes, five (41.7%) had smoking history, and nine (75.0%) had RP-ILD. Six (50.0%) of these 12 patients had elevated cardiac troponin I (cTnI) levels (normal range 0–0.056 μg/l), four patients (33.3%) had elevated creatine kinase MB (CK-MB) levels (normal range 0–3.6 μg/l), and N-terminal pro-brain natriuretic peptide (NT-proBNP) levels (normal range 0–125 pg/ml) were elevated in 10 (83.3%) patients on admission. All of the 12 patients had sinus tachycardia, and seven (58.3%) had low or inverted T waves in multiple leads. The most common abnormal findings were left atrial and/or ventricular enlargement noted in eight (66.7%) patients. Cardiac hypertrophy was noted in three (25.0%) patients. Diffuse ventricular wall dyskinesia was noted in six (50.0%) patients, and three (25.0%) of them developed obvious systolic dysfunction (left ventricular ejection fraction 20%, 45%, and 44% separately). Diastolic dysfunction was noted in seven (58.3%) patients. Both pericardial effusion and pulmonary hypertension were noted in two (16.7%) patients. Three patients (patients 2, 4, and 5) underwent CMR. Late gadolinium enhancement (LGE), the sign of myocarditis, was noted for all of them. Representative images are shown in **Figure 1**.

**Table 1:** Clinical features and cardiac examinations findings of anti-MDA5 Ab+ DM/CADM patients with myocardial involvement.

Patient	P1	P2	P3	P4	P5	P6	P7	P8	P9	P10	P11	P12
**Characteristics**												
Gender	M	F	F	M	F	M	F	F	F	M	M	M
Age, yrs	68	59	18	42	33	74	21	52	69	63	33	71
Disease duration, mos	2	21	6	3	4	2	12	3	1	3	1	4
Hypertension	+	–	–	–	–	–	–	–	–	+	–	–
Diabetes	+	–	–	–	–	–	–	–	+	–	–	–
Smoking	–	–	–	+	–	+	–	–	–	+	+	+
RP-ILD	+	–	+	–	–	+	+	+	+	+	+	+
**Laboratory results on admission**												
cTnI, μg/l	0.059	0.932	0.210	N	N	0.139	N	0.093	N	N	N	0.128
CK-MB, μg/l	2.8	3.1	6.0	3.3	19.2	2.0	0.6	5.4	1.2	1.4	1.5	5.9
NT-proBNP, pg/ml	2345	7971	2838	107	390	511	1334	1311	512	345	55	879
Cr, μmol/l	61	52	40	57	42	61	41	34	50	41	49	81
**ECG on admission**												
HR, bpm	108	120	109	107	112	104	102	100	118	120	105	93
Low/inverted T waves, lead	V2-V6	V4-V6	–	–	V3-V6	I aVL V2-V5	III aVL V4-V6	I, II, aVF V5-V6	–	–	–	I aVL V4-V6
**TTE findings**												
Atrial/ventricular Enlargement	–	+	+	–	–	+	–	+	+	+	+	+
Cardiac Hypertrophy	–	–	–	+	–	–	–	–	+	+	–	–
Ventricular wall Dyskinesia	+	+	+	+	+	–	+	–	–	–	–	–
Systolic Dysfunction	–	+	+	–	–	–	+	–	–	–	–	–
Diastolic Dysfunction	+	+	–	–	–	+	–	+	+	+	–	+
TTE parameters												
LVDd, mm	42	60	43	51	45	55	43	45	50	48	29	49
LVDs, mm	30	52	33	33	30	39	34	31	34	31	47	31
LVEF, %	56	20	45	65	64	55	44	57	60	65	68	66
LADs, mm	33	39	39	37	32	44	29	40	39	42	40	42
PWT, mm	9	9	6	12	7	9	7	9	11	11	8	9
IVST, mm	9	8	6	11	7	8	6	9	12	11	8	10
E/A	0.7	>2	1.2	1.2	1.2	0.7	1.2	0.6	0.7	0.5	0.9	0.7
**CMR**	NA	+	NA	+	+	NA	NA	NA	NA	NA	NA	NA

Statistical significance: p < 0.05.

+, yes; -, no; M, male; F, female; yrs, years; mos, months; N, normal; ECG, electrocardiography; TTE, transthoracic echocardiography; CMR, cardiac magnetic resonance imaging; RP-ILD, rapidly progressive interstitial lung disease; cTnI, cardiac troponin I; CK-MB, creatine kinase MB; NT-proBNP, N-terminal pro-B type natriuretic peptide; HR, heart rate; LVDd, left ventricular diameter at end diastole; LVDs, left ventricular internal dimension in systole; LVEF, left ventricular ejection fraction; LADs, left atrial internal dimension in systole; PWT, posterior LV wall thickness at end diastole; IVST, interventricular septal thickness at end diastole; E/A, E wave/A wave ratio, E wave, early diastolic filling velocity, A wave, atrial filling velocity; NA, not applicable.

Seven (58.3%) of the 12 patients with MI developed respiratory failure, and six (50.0%) patients developed heart failure (HF) during treatment. Besides bacterial infections (41.7%), opportunistic infections were common as well including five patients (41.7%) with cytomegalovirus (CMV) infection, two patients (16.7%) with Epstein–Barr virus (EBV) infection, two patients (16.7%) with Pneumocystis jirovecii infection, and two patients (16.7%) with fungal infection. All 12 patients received aggressive immunosuppressive therapy, including five patients with methylprednisolone pulse therapy (41.7%), six patients with triple-combined therapy with a high dose of glucocorticoids and two immunosuppressants (50.0%), eight patients with intravenous immunoglobulin therapy (IVIG) (66.7%), two patients with tocilizumab (16.7%), and one patient with plasmapheresis (8.3%). The complications, treatments, and short-term outcomes are summarized in **Table 2**. Eight (66.7%) of these 12 patients died, and the other four (33.3%) (patients 4, 5, 11, and 12) achieved remission. Of the 12 patients, three patients died of infection, one patient died of HF caused by myocarditis, and four patients died of respiratory failure caused by exacerbation of ILD. TTE was followed every 6 months after the discharge in two survival patients (patients 4 and 5). By the time of study data collection, both patients demonstrated normal cardiac structure and function by TTE. The TTE results are summarized in **Supplementary Table S2**. The follow-up times of patient 11 and patient 12 are less than 6 months, so the TTE results of these two patients are not available.

**Table 2:** Treatment and outcome of anti-MDA5 Ab+ DM/CADM patients with myocardial involvement.

Patient	P1	P2	P3	P4	P5	P6	P7	P8	P9	P10	P11	P12
**Complications**												
Respiratory failure	+	–	+	–	–	+	+	+	+	+	–	–
Heart failure	+	+	+	–	–	+	+	–	–	+	–	–
CMV infection	–	+	–	+	–	NA	NA	+	–	+	–	+
EBV infection	NA	+	+	–	–	NA	NA	–	–	–	–	–
PCP	–	–	+	–	–	–	–	–	+	–	–	–
Bacterial infection	+	+	+	–	–	–	–	–	+	–	–	+
Fungal infection	+	–	–	–	–	–	–	–	+	–	–	–
**Treatment**												
GC	+	+	+	+	+	+	+	+	+	+	+	+
Pulse therapy	+	+	+	+	+	–	–	–	–	–	–	–
Immunosuppressor	TAC CTX	CTX	–	TAC CTX	CTX	TAC	TAC MMF	–	TAC CTX	–	TAC CTX	TAC CTX
IVIG	+	–	+	+	–	–	+	+	–	+	+	+
Other	PE	–	–	–	–	–	–	–	–	–	T	T
Antiviral	+	+	–	+	–	–	–	+	–	+	–	+
Prophylactic SMZ	+	+	–	+	+	+	+	+	–	+	+	+
Therapeutic SMZ	–	–	+	–	–	–	–	–	+	–	–	–
Antibiotics	+	+	+	–	–	+	+	+	+	+	–	+
Antifungal	+	–	–	–	–	–	–	–	+	–	–	–
**Short-term outcome**												
Deceased	+	+	+	–	–	+	+	+	+	+	–	–
Cause of death	INF	HF	INF	NA	NA	RF	RF	RF	INF	RF	NA	NA

+, yes; -, no; NA, not applicable; CMV, cytomegalovirus; EBV, Epstein–Barr virus; PCP, pneumocystis pneumonia; GC, glucocorticoids; IVIG, intravenous immunoglobulin; SMZ, sulfamethoxazole; CTX, cyclophosphamide; TAC, tacrolimus; MMF, mycophenolate mofetil; PE, plasmapheresis; T, tocilizumab; INF, infection; HF, heart failure; RF, respiratory failure.

### Comparison of clinical manifestations between patients with or without myocardial involvement

We assigned these 76 patients into MI group (n = 12) and non-MI group (n = 64) according to whether they were complicated with MI. We compared the clinical features between two groups (**Table 3**). No significant differences between groups were noted for demographic features, comorbidities, and dermatomyositis-related characteristics, except patients in the MI group who were significantly more likely to present with dysphagia. With regard to laboratory findings, levels of creatine kinase (CK), lactate dehydrogenase (LDH), and NT-proBNP and percentages of patients with elevated cTnI and elevated CK-MB were significantly higher in the MI group when compared with the non-MI group. Myocardial enzymes are the key markers of myocardial injury, and NT-proBNP is useful for risk stratification in HF. According to the European Society of Cardiology (ESC) guideline, independent of age, an NT-proBNP concentration >600 pg/ml provides excellent positive predictive value for chronic HF (14). The percentage of patients with NT-proBNP >600 pg/ml was significantly higher in the MI group when compared with the non-MI group (45.5% vs. 5.7%, *p* = 0.005). The MI group had a lower level of plasma albumin (ALB) but a higher level of peripheral blood white blood cells (WBCs). Serum ferritin ≥1,500 ng/ml was identified as an independent predictor for RP-ILD and a poor prognostic risk factor of anti-MDA5 Ab+ DM/CADM patients (15). No significant differences were observed in the levels of ferritin and the percentages of patients with ferritin ≥1,500 ng/ml between two groups. Similar to ferritin, there were no differences between the two groups in terms of inflammatory indicators including erythrocyte sedimentation rate (ESR) and C-reactive protein (CRP). Both groups were vulnerable to opportunistic infection with comparable prevalence of CMV, EBV, Pneumocystis jirovecii, bacteria, and fungi. The mortality of the MI group was significantly higher than the non-MI group (66.7% vs. 26.6%, *p* = 0.009).

**Table 3:** Comparison of characteristics between patients with and without myocardial involvement.

Variables	MI group n = 12	Non-MI group n = 64	*p* value
**Demographics**			
Gender, female, n (%)	6 (50.0%)	43 (67.2%)	0.254
Age at diagnosis, yrs	55.5 (33.0-68.5)	54.0 (43.0-59.5)	0.679
Disease duration, mos	3.0 (2.0-5.0)	4.0 (2.5-9.0)	0.336
On-admission BMI, kg/m^2^	21.9 (20.1-25.3)	22.0 (20.0-24.1)	0.680
Weight lost in the last 3 months, kg	4.0 (0.0-12.5)	5.0 (0.0-10.0)	0.659
Hypertension, n (%)	2 (16.7%)	14 (21.9%)	0.678
Diabetes, n (%)	2 (16.7%)	11 (17.2%)	0.965
Smoking, n (%)	5 (41.7%)	12 (18.8%)	0.080
**Clinical manifestations**			
Skin ulcer, n (%)	3 (25.0%)	12 (18.8%)	0.626
Digits vasculitis, n (%)	3 (25.0%)	21 (32.8%)	0.587
Muscle pain, n (%)	5 (41.7%)	21 (32.8%)	0.553
Muscle weakness, n (%)	5 (41.7%)	14 (21.9%)	0.146
Dysphagia, n (%)	6 (50.0%)	13 (20.3%)	**0.029**
Arthralgia/arthritis, n (%)	7 (58.3%)	38 (59.3%)	0.946
RP-ILD, n (%)	9 (75.0%)	29 (45.3%)	0.054
Pneumothorax/pneumomediastinum, n (%)	2 (16.7%)	13 (20.3%)	0.767
PaO_2_ <60 mmHg, n (%)	7 (58.3%)	25 (39.1%)	0.215
**On-admission laboratory features**			
Ferritin, ng/ml	1792.0 (1118.5-3041.0)	1309.0 (731.0-1975.5)	0.103
Ferritin >1,500 ng/ml, n (%), n = 11|59	6 (54.5%)	23 (39.0%)	0.336
ESR, mm/h	32.0 (20.0-55.5)	38.0 (19.0-54.0)	0.924
CRP, mg/l	10.3 (3.0-31.7)	3.1 90.6-13.4)	0.077
CK, U/l	297.0 (179.0-520.0)	85.0 (49.0-228.0)	**0.010**
LDH, U/l	561.5 (376.0-711.0)	343.0 (278.0-512.0)	**0.045**
AST, U/l	107.0 (68.0-149.0)	58.5 (37.0-92.0)	0.060
ALT, U/l	76.0 (56.0-123.5)	59.0 (38.5.0-89.5)	0.198
GGT, U/l	186.0 (85.0-550.0)	109.0 (49.0-201.0)	0.108
ALP, U/l	112.5 (90.0-173.0)	74.5 (62.0-121.0)	0.057
ALB, g/l	28.3 ± 7.3	31.9 ± 5.0	**0.040**
Cr, μmol/l	49.5 (40.5-59.0)	56.0 (48.0-66.0)	0.087
WBC, 10^9/L	7.1 (6.2-8.1)	5.7 (3.9-7.5)	**0.019**
NEUT, 10^9/L	5.5 (4.9-6.8)	4.1 (2.8-6.3)	0.082
LYM, 10^9/L	0.8 (0.4-1.1)	0.5 (0.4-1.0)	0.069
NLR	8.1 (4.9-18.8)	5.9 (3.6-14.4)	0.333
HGB, g/l	115.4 ± 29.7	120.3 ± 19.1	0.595
PLT, 10^9/L	172.0 (98.5-204.0)	175.0 (126.0-225.5)	0.409
Elevated cTnI, n (%), n = 12|55	6 (50.0%)	4 (7.2%)	**0.004**
Elevated CK-MB, n (%), n = 12|50	3 (25.0%)	8 (16.0%)	**0.021**
NT-proBNP, pg/ml	512.0 (367.5-1322.5)	118.0 (66.0-233.5)	**<0.001**
NT-BNP >600 pg/ml, n (%), n = 11|53	5 (45.5%)	3 (5.7%)	**0.005**
**Infection**			
CMV infection, n (%), n = 10|55	5 (50.0%)	21 (38.2%)	0.764
EBV infection, n (%), n = 9|54	2 (22.2%)	3 (5.6%)	0.247
PCP, n (%), n = 12|59	2 (16.7%)	11 (18.6%)	0.405
Bacterial infection, n (%), n = 12|57	5 (41.7%)	16 (28.1%)	0.304
Fungal infection, n (%), n = 12|58	2 (16.7%)	4 (6.9%)	0.204
Short-term outcome			
Deceased, n (%)	8 (66.7%)	17 (26.6%)	**0.009**

Continuous variables are presented as mean ± SD if normal distribution and median (interquartile range) if skewed distribution. Categorical variables were presented as n (%). Statistical significance: p < 0.05.

yrs, years; mos, months; BMI, body mass index; RP-ILD, rapidly progressive interstitial lung disease; ESR, erythrocyte sedimentation rate; CRP, C-reactive protein; CK, creatine kinase; LDH, lactate dehydrogenase; AST, aspartate aminotransferase; ALT, alanine aminotransferase; GGT, γ-glutamyl transferase; ALP, alkaline phosphatase; ALB, albumin; Cr, creatinine; WBC, white blood cell; NEUT, neutrophil; LYM, lymphocyte; NLR, neutrophil-to-lymphocyte ratio; HGB, hemoglobin; PLT, platelet; cTnI, cardiac troponin I; CK-MB, creatine kinase MB; NT-proBNP, N-terminal pro-B type natriuretic peptide; CMV, cytomegalovirus; EBV, Epstein–Barr virus; PCP, pneumocystis pneumonia.

We selected the variables with significant differences between MI and non-MI groups for logistic regression analysis, to identify risk factors for the occurrence of MI. Univariate logistic regression analysis showed four factors associated with MI in anti-MDA5 Ab+ DM/CADM patients, which included three risk factors containing dysphagia (OR 3.923, 95% CI 1.085, 14.181, *p* = 0.037), increased peripheral WBCs (OR 1.201, 95% CI 1.003, 1.438, *p* = 0.046), and NT-proBNP >600 pg/ml (OR 18.333, 95% CI 1.508, 222.875, *p* = 0.022), and one protective factor plasma ALB (OR 0.892, 95% CI 0.796, 0.999, *p* = 0.048) (**Table 4**). Due to the small sample size of the MI group, multivariate analysis was not performed.

**Table 4:** Logistic regression analysis of associated factors for myocardial involvement of anti-MDA5 Ab+ DM/CADM patients.

Risk factors	*p*	Univariate OR	95% CI
Dysphagia	**0.037**	3.923	1.085, 14.181
CK	0.242	1.000	1.000, 1.001
LDH	0.230	1.001	0.999, 1.004
ALB	**0.048**	0.892	0.796, 0.999
WBC	**0.046**	1.201	1.003, 1.438
Elevated cTnI	0.534	0.438	0.032, 5.926
Elevated CK-MB	0.467	0.571	0.126, 2.586
NT-proBNP >600 pg/ml	**0.022**	18.333	1.508, 222.875

Statistical significance: p < 0.05.

CK, creatine kinase; LDH, lactate dehydrogenase; ALB, albumin; WBC, white blood cell; cTnI, cardiac troponin I; CK-MB, creatine kinase MB; NT-proBNP, N-terminal pro-B type natriuretic peptide.

### Comparison of clinical manifestations between deceased and survival patients

The clinical manifestations between deceased and survival patients are shown in **Table 5**. Patients in the deceased group had a shorter disease duration (3.0 vs. 6.0 months, *p* = 0.014) and a higher smoking rate (36.0% vs. 15.7%, *p* = 0.046). There were no significant differences noted for age, gender, body mass index (BMI), body weight loss, comorbidities, and dermatomyositis-related characteristics. Percentages of dysphagia (52.0% vs. 23.5%, *p* = 0.013), MI (32.0% vs. 7.8%, *p* = 0.007), RP-ILD (88.0% vs. 31.4%, *p* < 0.001), and respiratory failure (84.0% vs. 21.6%, *p* < 0.001) were significantly higher in the deceased group when compared with the survival group. Levels of inflammatory indicators including ferritin (1902.5 vs. 1190.0 mg/l, *p* < 0.001), ESR (50.0 vs. 27.0 mm/h, *p* = 0.017), and CRP (14.3 vs. 2.3 mg/l, *p* = 0.001) were higher in the deceased group than in the survival group. The percentage of patients with ferritin ≥ 1,500 ng/ml was significantly higher in the deceased group (68.2% vs. 29.2%, *p* = 0.002). The deceased group also had higher levels of CK (233.0 vs. 152.0 U/l, *p* = 0.016), LDH (637.4 vs. 311.0 U/l, *p* < 0.001), and alkaline phosphatase (ALP) (107.0 vs. 76.0 U/l, *p* = 0.027). The levels of leukocyte fractions, including neutrophils and lymphocytes, were significantly different between the two groups. The deceased group had a significantly higher neutrophil-to-lymphocyte ratio (NLR) than the survival group (13.3 vs. 5.1, *p* = 0.004). Percentages of patients with elevated cTnI (33.3% vs. 4.6%, *p* = 0.006) and with NT-proBNP >600 pg/ml (20.8% vs. 7.5%, *p* = 0.029) were significantly different between the two groups. In addition, the deceased group was more likely to be complicated with pneumocystis pneumonia (PCP) (36.3% vs. 10.2%, *p* = 0.014) but less likely to receive treatment of triple therapy including glucocorticoids, cyclophosphamide and tacrolimus (28.0% vs. 66.7%, *p* = 0.001), and tocilizumab (12.0% vs. 33.3%, *p* = 0.037), although IVIG was administered more frequently (84.0% vs. 51.0%, *p* = 0.037).

**Table 5:** Comparison of characteristics between deceased group and survival group.

Variables	Deceased group n = 25	Survival group n = 51	*p* value
**Demographics**			
Gender, female, n (%)	14 (56.0%)	35 (68.6%)	0.280
Age at diagnosis, yrs	57.0 (50.5-63.0)	57.0 (50.5-63.0)	0.104
Disease duration, mos	3.0 (2.0-4.0)	6.0 (3.0-11.5)	**0.014**
On-admission BMI, kg/m^2^	21.9 (19.8-24.7)	21.7 (20.7-24.4)	0.349
Weight lost in the last 3 months, kg	5.0 (0.0-10.0)	5.0 (0.0-9.0)	0.286
Hypertension, n (%)	6 (24.0%)	10 (19.6%)	0.659
Diabetes, n (%)	3 (12.0%)	10 (19.6%)	0.408
Known CHD, n (%)	2 (8.0%)	1 (2.0%)	0.204
Smoking, n (%)	9 (36.0%)	8 (15.7%)	**0.046**
**Clinical manifestations**			
Skin ulcer, n (%)	3 (12.0%)	12 (23.5%)	0.379
Digits vasculitis, n (%)	4 (16.0%)	20 (39.2%)	0.075
Muscle pain, n (%)	10 (40.0%)	16 (31.4%)	0.456
Muscle weakness, n (%)	13 (52.0%)	29 (56.9%)	0.689
Dysphagia, n (%)	13 (52.0%)	12 (23.5%)	**0.013**
Arthralgia/arthritis, n (%)	14 (56.0%)	31 (60.8%)	0.690
MI, n (%)	8 (32.0%)	4 (7.8%)	**0.007**
RP-ILD, n (%)	22 (88.0%)	16 (31.4%)	**<0.001**
Pneumothorax/pneumomediastinum, n (%)	7 (28.0%)	8 (15.7%)	0.205
PaO_2_ <60 mmHg, n (%)	21 (84.0%)	11 (21.6%)	**<0.001**
**On-admission laboratory features**			
Ferritin, ng/ml	1,902.5 (1464.0-4089.0)	1,190.0 (748.5-1840.5)	**<0.001**
Ferritin >1,500 ng/ml, n (%), n = 22|48	15 (68.2%)	14 (29.2%)	**0.002**
ESR, mm/h	50.0 (29.5-62.0)	27.0 (15.0-48.0)	**0.017**
CRP, mg/l	14.3 (3.4-71.4)	2.3 (0.5-5.7)	**0.001**
CK, U/l	233.0 (142.5-371.0)	152.0 (37.0-279.0)	**0.016**
LDH, U/l	637.4 (463.0-755.0)	311.0 (273.0-385.0)	**<0.001**
AST, U/l	81.5 (51.0-216.0)	61.5 (40.3-107.8)	0.054
ALT, U/l	56.5 (27.0-203.3)	71.0 (41.3-107.8)	0.937
GGT, U/l	192.0 (62.8-428.0)	110.0 (75.8-271.3)	0.076
ALP, U/l	107.0 (71.3-187.5)	76.0 (61.0-116.5)	**0.027**
ALB, g/l	27.6 ± 6.3	33.2 ± 4.0	**<0.001**
Cr, μmol/l	62.3 (40.0-61.0)	56.0 (948.5-66.0)	0.224
WBC, 10^9/L	6.6 (5.4-8.1)	5.7 (3.8-7.2)	**0.029**
NEUT, 10^9/L	6.7 (4.6-7.3)	4.0 (2.8-5.8)	**0.003**
LYM, 10^9/L	0.6 (0.3-0.7)	0.7 (0.4-1.1)	**0.028**
NLR	13.3 (6.4-19.5)	5.1 (3.5-9.6)	**0.004**
HGB, g/l	120.0 ± 27.5	121.7 ± 14.4	0.670
PLT, 10^9/L	170.0 ± 75.7	177.7 ± 65.1	0.687
Elevated cTnI, n (%), n = 24|43	8 (33.3%)	2 (4.6%)	**0.006**
Elevated CK-MB, n (%), n = 23|39	5 (21.7%)	6 (18.2%)	0.211
NT-proBNP, pg/ml	200.5 (103.5-511.8)	132.0 (72.5-271.3)	0.114
NT-BNP >600 pg/ml, n (%), n = 24|40	5 (20.8%)	3 (7.5%)	**0.029**
**Infection**			
CMV infection, n (%), n = 20|45	8 (40.0%)	18 (40.0%)	0.632
PCP, n (%), n = 22|49	8 (36.3%)	5 (10.2%)	**0.014**
Bacterial infection, n (%), n = 23|46	10 (43.5%)	11 (23.9%)	0.240
Fungal infection, n (%), n = 22|48	3 (13.6%)	3 (6.3%)	0.407
**Medications**			
Pulse therapy, n (%)	4 (16.0%)	7 (13.7%)	0.793
CTX, n (%)	4 (16.0%)	4 (7.8%)	0.290
TAC, n (%)	1 (4.0%)	7 (13.7%)	0.162
CTX+TAC, n (%)	7 (28.0%)	34 (66.7%)	**0.001**
IVIG, n (%)	21 (84.0%)	26 (51.0%)	**0.037**
Steroid + DMARDs + tocilizumab, n (%)	3 (12.0%)	17 (33.3%)	**0.037**

Continuous variables are presented as mean ± SD if normal distribution and median (interquartile range) if skewed distribution. Categorical variables were presented as n (%). Statistical significance: p < 0.05.

yrs, years; mos, months; BMI, body mass index; CHD, coronary heart disease; MI, myocardial involvement; RP-ILD, rapidly progressive interstitial lung disease; ESR, erythrocyte sedimentation rate; CRP, C-reactive protein; CK, creatine kinase; LDH, lactate dehydrogenase; AST, aspartate aminotransferase; ALT, alanine aminotransferase; GGT, γ-glutamyl transferase; ALP, alkaline phosphatase; Cr, creatinine; WBC, white blood cell; NEUT, neutrophil; LYM, lymphocyte; NLR, neutrophil-to-lymphocyte ratio; HGB, hemoglobin; PLT, platelet; cTnI, cardiac troponin I; CK-MB, creatine kinase MB; NT-proBNP, N-terminal pro-B type natriuretic peptide; CMV, cytomegalovirus; PCP, pneumocystis pneumonia; CTX, cyclophosphamide; TAC, tacrolimus; IVIG, intravenous immunoglobulin; DMARDs, disease-modifying anti-rheumatic drugs.

Univariate logistic regression analysis showed that there were 16 variables associated with death at the level of *p* < 0.05 (**Supplementary Table S3**). Due to the small sample size of the deceased group, it is inappropriate to introduce all 16 variables into the multivariate analysis. Increased levels of LDH, ferritin, CRP, ESR, and NLR but decreased levels of ALB have been reported to be poor prognostic factors of PM/DM or PM/DM-associated ILD (16–19). Especially, LDH and ferritin are established as serum biomarkers related to prognosis in anti-MDA5 Ab+ DM (20). Factors including myocardial injury, exacerbation of interstitial lung disease, and infection are the common causes of clinical death, which are also the focus of this study. These three complications can lead to changes in the levels of serum markers. We removed redundant information and selected three complications, namely, MI, RP-ILD, and PCP, for multivariate regression analysis. Multivariate logistic regression analysis revealed that both MI (OR 5.984, 95% CI 1.174, 30.496, *p* = 0.031) and RP-ILD (OR 11.875, 95% CI 2.796, 50.411, *p* = 0.001) were independent risk factors for the death of these anti-MDA5 Ab+ DM/CADM patients (**Table 6**).

**Table 6:** Logistic regression analysis of associated factors for the death of anti-MDA5 Ab+ DM/CADM patients.

Risk factors	*p*	Univariate OR	95% CI	*p*	Multivariate OR	95% CI
MI	0.011	5.529	1.474, 20.745	0.031	5.984	1.174, 30.496
RP-ILD	<0.001	16.042	4.186, 61.478	0.001	11.875	2.796, 50.411
PCP	0.013	5.029	1.414, 17.887	0.087	7.502	0.746, 75.437

MI, myocardial involvement; RP-ILD, rapidly progressive interstitial lung disease; PCP, pneumocystis pneumonia.

## Discussion

In this single-center, retrospective cohort study, we, for the first time, reported MI in a large group of anti-MDA5 Ab+ DM/CADM patients. Myocardial involvement is not rare in anti-MDA5 Ab+ DM/CADM patients and is an independent risk factor for unfavorable outcomes in these patients.

IIM is a heterogeneous group of diseases in which the heart is one of the most severe organ involvements due to myocardial inflammation and ventricular dysfunction. Manifestations of cardiac involvement such as dyspnea on exertion, palpitation, and chest pain are non-specific and subtle. The differences in the definition of cardiac involvement and in the observed population lead to a large variety of prevalence of cardiac involvement from 9% to 72% in IIM (5, 21). Recently, MSAs have been increasingly used to subcategorize IIM patients, while the relationship between MSAs and cardiac involvement still needs to be clarified. For example, anti-signal recognition particle (anti-SRP) antibodies were initially considered to be unrelated to increased risk of cardiac involvement (22). However, according to a recent multicenter study on adult IIM patients, the percentage of positive anti-SRP antibodies was significantly higher in the MI group than in the control group (23). It has been generally assumed that anti-MDA5 Ab+ DM/CADM is accompanied by mild or absent muscle involvement; however, a previous study indicated that half of their anti-MDA5 Ab+ DM/CADM patients could present with cardiac manifestations (24). Unfortunately, detailed information was not provided in this study. As mentioned above, only three cases of anti-MDA5 Ab+ DM/CADM patients with severe myocardial defects have been reported so far. In the present cohort study, we are able to demonstrate that MI is not rare in anti-MDA5 Ab+ DM/CADM patients but with a prevalence of 15.8%. Cardiac involvement was documented to be responsible for deaths in 10%–20% PM patients (21). In our study, we also firstly reported that MI was an independent prognostic factor for the death of anti-MDA5 Ab+ DM/CADM patients in addition to the already well-known prognostic factor RP-ILD.

In our study, ventricular wall dyskinesia was observed in 50% patients who have MI, and three of them had a disease duration longer than 6 months and were complicated with severe systolic dysfunction presenting as significantly decreased left ventricular ejection fraction (LVEF). This phenomenon is consistent with two former reported cases (a 55-year-old man with a 7-month disease duration and a 48-year-old man with a 6-month disease duration) with a severe LVEF decline (6, 8). The results suggest that the MI of anti-MDA5 Ab+ DM/CADM predisposes patients to severe cardiac systolic dysfunction along with disease duration. In addition, seven (58.3%) patients had unexplained cardiac diastolic dysfunction as well, which we believe may be a sign of MI and merited further detailed evaluation for subclinical myocardial defects. More than half of patients with MI showed abnormality in T waves of multiple leads in our study. This result is consistent with the findings of a recent study (9). Changes in T waves in ECG of anti-MDA5 Ab+ DM/CADM patients is supposed to be another early sign for the occurrence of MI.

Dysphagia is a hallmark of IIM, whereas patients with anti-MDA5 Ab+ were considered less likely to develop dysphagia in a previous study (24). Our patients with MI were more likely to be concomitant with dysphagia. Mechanisms of dysphagia refer to both skeletal-muscle and smooth-muscle abnormalities, which may be the shared mechanism of MI. Therefore, dysphagia is supposed to be a red-flag sign of the occurrence of MI. NT-proBNP was frequently elevated in MI anti-MDA5 Ab+ patients and was associated with death. An elevated NT-proBNP level is supposed to be another red-flag sign for the occurrence of MI. A reasonable cutoff value is needed to be verified in a larger cohort. There were significant differences in leukocyte levels between MI and non-MI groups. Increased NLR was reported to be an independent risk factor for RP-ILD of anti-MDA5 Ab+ DM/CADM patients and a poor prognostic factor for IIM patients (16, 25). In our study, the deceased group had a significantly higher NLR, which is consistent with those reported. A number of factors like infection and drugs can influence the levels and fractions of leukocytes. We were unable to conclude that leukocytes contribute to the pathogenesis of IIM.

MDA5 is an interferon (IFN)-inducible host cell DExD/H box helicase located in the cytosol and plays a crucial role in triggering the innate immune system to defense against viruses through recognizing viral double-stranded RNA and activates transcription of type I IFN genes (26, 27). Previous studies suggest that MDA5 is likely to play a crucial role in protecting the heart from acute viral myocarditis. MDA5-knockout mice are more susceptible to encephalomyocarditis virus (EMCV) infection and develop lethal myocardial injury (28). Similar results have been reported in coxsackievirus B3 (CVB3)-infected MDA5-knockout mice (29). In contrast, the cardiac-specific overexpression of MDA5 attenuates EMCV-induced cardiac myocyte apoptosis and protects mice from myocarditis and heart dysfunction (28). Nevertheless, the exact roles of MDA5 and anti-MDA5 antibody in human autoimmune myocarditis need further clarifying.

The limitations of this study include the following points. First, the nature of the study design (retrospective single-center study) increased the risk of selective bias. Hospitalized patients were more serious, which might lead to overestimation of the prevalence and mortality of MI in anti-MDA5 Ab+ DM/CADM patients. Second, cardiac involvement refers to a variety of areas including conduction system, pericardium, and coronary artery other than myocardium. Our study focused on myocardium and thus did not reveal the overall picture of cardiac involvement in anti-MDA5 Ab+ DM/CADM patients. Third, due to the rarity of previous reports on MI in anti-MDA5 Ab+ DM/CADM patients, the majority of patients only completed myocardial enzyme detection, ECG, and TTE, while CMR was usually lacking, let alone myocardial biopsy. Multicenter studies and myocardial biopsy are needed to accurately assess the morbidity and pathogenesis of MI in anti-MDA5 Ab+ DM/CADM patients.

In conclusion, our work described the precise clinical characteristics of MI in anti-MDA5 Ab+ DM/CADM patients. Myocardial involvement is an independent risk factor for the mortality of anti-MDA5 Ab+ DM/CADM patients. Due to the subclinical nature of heart involvement and a poor prognosis if treatment is delayed, we underline the importance of cardiac abnormalities screening in anti-MDA5 Ab+ DM/CADM patients at the time of diagnosis and during follow-up.

## Data availability statement

The original contributions presented in the study are included in the article/**Supplementary Material**. Further inquiries can be directed to the corresponding authors.

## Ethics statement

The studies involving human participants were reviewed and approved by the medical ethics committee of Peking Union Medical College Hospital (approval number: S-K1997). Written informed consent for participation was not required for this study in accordance with the national legislation and the institutional requirements.

## Author contributions

SZ, JL, YW, and QW conceptualized the study. SZ, JL, CW, YTL, YXL, JZ, and DX performed the data collection. SZ, JL, XT, YZ, and ML performed the data analysis. SZ and JL drafted the manuscript. YW, QW, and XZ revised the manuscript. All authors provided critical comments and a final consent to the submission. All authors contributed to the article and approved the submitted version.

## Funding

This study was supported by CAMS Innovation Fund for Medical Sciences (CIFMS) (2021-I2M-1-005 and 2019-I2M-2-008), the Beijing Municipal Science & Technology Commission (Z201100005520025), and the National Natural Science Foundation of China (81471615, 81601430, and 81873891).

## Conflict of interest

The authors declare that the research was conducted in the absence of any commercial or financial relationships that could be construed as a potential conflict of interest.

## Publisher’s note

All claims expressed in this article are solely those of the authors and do not necessarily represent those of their affiliated organizations, or those of the publisher, the editors and the reviewers. Any product that may be evaluated in this article, or claim that may be made by its manufacturer, is not guaranteed or endorsed by the publisher.
