# Supplementary material for: Myocardial involvement is not rare in anti-melanoma differentiation-associated gene 5 antibody-positive dermatomyositis/clinically amyopathic dermatomyositis: a retrospective study
Source: Front Immunol. 2022 Aug 2;13:928861. doi: 10.3389/fimmu.2022.928861 (PMC9379921; doi:10.3389/fimmu.2022.928861)
Supplement: Supplementary file 1 [file Table_1.docx]

**Supplementary Table S1. Overview of cardiac examinations underwent by anti-MDA5 Ab+ DM/CADM patients.**

|  | **Total**  **n=100** |
| --- | --- |
| **Patients underwent myocardial enzyme testing on admission** | |
| cTnI, n (%) | 90 (90.0%) |
| Elevated cTnI, n (%) | 10 (11.1%) |
| CK-MB, n (%) | 83 (83.0%) |
| Elevated CK-MB, n (%) | 12 (14.5%) |
| NT-proBNP, n (%) | 86 (86.0%) |
| Elevated NT-proBNP, n (%) | 52 (60.5%) |
| **Patients underwent ECG on admission, n (%)** | 97 (97.0%) |
| ST-T changes, n (%) | 20 (20.6%) |
| Arrhythmias, n (%) | 2 (2.1%) |
| Left ventricular high voltage, n (%) | 1 (1.0%) |
| **Patients underwent TTE, n (%)** | 76 (76.0%) |
| Atrial/Ventricular Enlargement, n (%) | 12 (15.8%) |
| Cardiac Hypertrophy, n (%) | 4 (5.3%) |
| Ventricular Wall Dyskinesia, n (%) | 5 (6.6%) |
| Systolic Dysfunction, n (%) | 4 (5.3%) |
| Diastolic Dysfunction, n (%) | 31 (40.8%) |
| Pericardial Effusion, n (%) | 8 (10.5%) |
| Pulmonary Hypertension, n (%) | 2 (2.6%) |
| **Patients underwent CMR, n (%)** | 3 (3.0%) |
| Diagnosis of myocarditis, n (%) | 3 (100%) |

Data are presented as n (%).

ECG, electrocardiography; TTE, transthoracic echocardiography; CMR, cardiac magnetic resonance imaging; cTnI, cardiac troponin I; CK-MB, creatine kinase MB; NT-proBNP, N-terminal pro-B type natriuretic peptide.

**Supplementary Table S2. Follow-Up of TEE changes of anti-MDA5 Ab+ DM/CADM patients with myocardial involvement.**

| **Patient** | **P4** | | | **P5** | |
| --- | --- | --- | --- | --- | --- |
| **Follow-Up duration, mos** | **Baseline** | **6** | **12** | **Baseline** | **6** |
| **TTE diagnosis** | | | | | |
| Atrial/Ventricular Enlargement | - | - | - | - | - |
| Cardiac Hypertrophy | + | - | - | - | - |
| Ventricular Wall Dyskinesia | + | - | - | + | - |
| Systolic Dysfunction | - | - | - | - | - |
| Diastolic Dysfunction | - | - | - | - | - |
| Pericardial Effusion | + | - | - | - | - |
| Pulmonary Hypertension | - | - | - | - | - |
| **TTE Parameters** | | | | | |
| LVDd, mm | 51 | 48 | 47 | 45 | 49 |
| LVDs, mm | 33 | 31 | 31 | 30 | 29 |
| LVEF, % | 65 | 64 | 65 | 64 | 71 |
| LADs, mm | 37 | 36 | 36 | 32 | 31 |
| PWT, mm | 12 | 8 | 7 | 7 | 7 |
| IVST, mm | 11 | 7 | 7 | 7 | 7 |
| E/A | 1.2 | 1.2 | 1.2 | 1.2 | 1.3 |

+, yes; -, no; mos, months; TTE, transthoracic echocardiography; LVDd, left ventricular diameter at end-diastole; LVDs, left ventricular internal dimension in systole; LVEF, left ventricular ejection fraction; LAD, left atrial dimension; PWT, posterior LV wall thickness at end-diastole; IVST, interventricular septal thickness at end diastole; E/A, E wave/A wave ratio, E wave, early diastolic filling velocity, A wave, atrial filling velocity.

**Supplementary Table S3. Univariate logistic regression analysis of associated factors for the death of anti-MDA5 Ab+ DM/CADM patients.**

| **Risk Factors** | ***P*** | **Univariate**  **OR** | **95% CI** |
| --- | --- | --- | --- |
| Disease duration | 0.154 | 0.932 | 0.846, 1.021 |
| Smoking | 0.051 | 3.023 | 0.994, 9.192 |
| Dysphagia | 0.015 | 3.521 | 1.273, 9.734 |
| MI | 0.011 | 5.529 | 1.474, 20.745 |
| RP-ILD | ＜0.001 | 16.042 | 4.186, 61.478 |
| PaO2<60mmHg | ＜0.001 | 19.091 | 5.413, 67.329 |
| Ferritin>1500 ng/ml | 0.003 | 5.204 | 1.746, 15.508 |
| ESR | 0.024 | 1.022 | 1.003, 1.041 |
| CRP | 0.006 | 1.029 | 1.008, 1.051 |
| CK | 0.164 | 1.001 | 1.000, 1.002 |
| LDH | ＜0.001 | 1.007 | 1.004, 1.010 |
| ALP | 0.026 | 1.010 | 1.001, 1.019 |
| ALB | ＜0.001 | 0.777 | 0.677, 0.892 |
| WBC | 0.022 | 1.228 | 1.030, 1.463 |
| NEUT | 0.006 | 1.340 | 1.090, 1.649 |
| LYM | 0.060 | 0.353 | 0.119, 1.011 |
| NLR | 0.029 | 1.041 | 1.004, 1.080 |
| Elevated cTnI | 0.009 | 3.317 | 2.394, 27.007 |
| NT-proBNP>600 pg/ml | 0.022 | 18.333 | 1.508, 222.875 |
| PCP | 0.013 | 5.029 | 1.414, 17.887 |

Statistical significance: *p* < 0.05.

BMI, body mass index; CHD, Coronary heart disease; MI, myocardial involvement; RP-ILD, rapidly progressive interstitial lung disease; ESR, erythrocyte sedimentation rate; CRP, C-reactive protein; CK, creatine kinase; LDH, lactate dehydrogenase; ALP, alkaline phosphatase; ALB, albumin; WBC, white blood cell; NEUT, neutrophil; LYM, lymphocyte; NLR, neutrophil-to-lymphocyte ratio; cTnI, cardiac troponin I; NT-proBNP, N-terminal pro-B type natriuretic peptide; PCP, pneumocystis pneumonia.
